# Artificial intelligence meets body sense: task-driven neural networks reveal computational principles of the proprioceptive pathway

**DOI:** 10.1038/s41392-024-01870-9

**Published:** 2024-07-08

**Authors:** Leonard E. van Dyck, Frank Bremmer, Katharina Dobs

**Affiliations:** 1 Department of Psychology, Justus Liebig University Giessen, Giessen, Germany; 2 Max Planck Institute for Human Cognitive and Brain Sciences, Leipzig, Germany; 3 Center for Mind, Brain and Behavior, Universities of Marburg, Giessen, and Darmstadt, Marburg, Germany; 4 Department of Neurophysics, Philipps-Universität Marburg, Marburg, Germany

**Keywords:** Neuroscience, Computational biology and bioinformatics

In a recent study published in *Cell*, Marin Vargas and Bisi et al.^1^ present an innovative approach to unravel the computational principles underlying proprioceptive processing in non-human primates. Their findings showcase the utility of task-driven modeling in advancing neuroscience and offer translational potential by providing seminal insights into the goals and mechanisms by which the brain encodes body position and movements.

Proprioception allows us to perceive the position and movement of our body parts and is crucial for motor control and coordination, such as when reaching for a light switch in the dark. Proprioceptive signals originate from specialized mechanoreceptors in muscles, tendons, and joints, and travel through the dorsal column-medial lemniscus pathway. Within this pathway, the cuneate nucleus (CN) plays a pivotal role in processing sensory information from the upper limbs and trunk. It then directs this information through the thalamus to reach both the primary (S1) and secondary somatosensory cortices. In these cortical areas, proprioceptive signals are integrated with other sensory information, typically shaping our perception of body position and movement unconsciously. Despite this understanding, the precise mechanisms involved in proprioception are still unclear. In particular, what are the computational goals of the proprioceptive pathway, and how does it encode proprioceptive signals to support these goals?

Marin Vargas and Bisi et al. address these questions through advanced computational modeling. Artificial neural networks have become powerful tools for studying neural processing across both sensory and motor pathways.^2,3^ These models not only achieve high predictive accuracy but also offer deep insights into the computational principles underlying neural responses. By training these networks on various tasks and comparing the learned representations to actual neural activity, researchers can explore the specific functions that these neural responses may serve, potentially unlocking new understandings of neural processing mechanisms.^4^

**Fig. 1:**
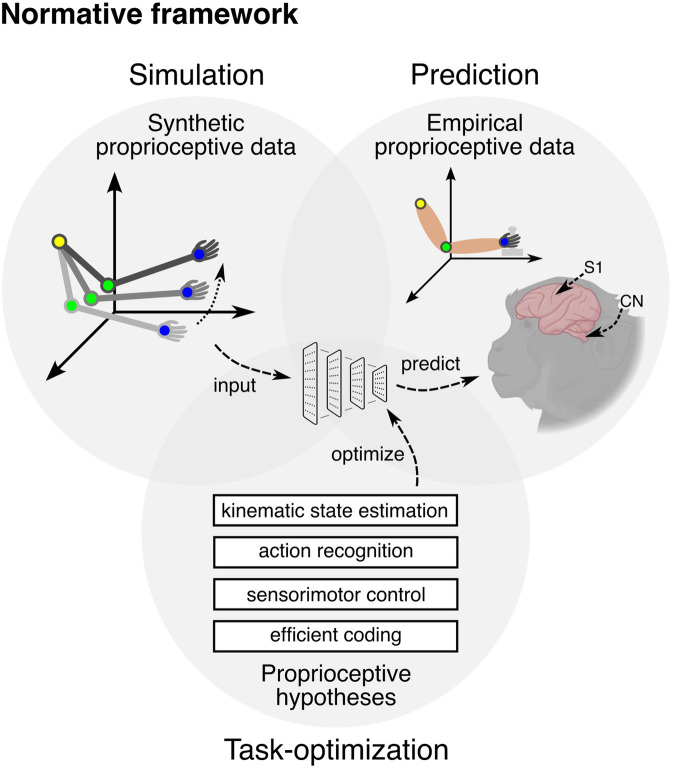
Normative framework. The normative framework to reveal the computational principles of the proprioceptive pathway. Neural network models were trained on a large-scale dataset of synthetic proprioceptive data, each optimized for specific hypothesized tasks, and tested by predicting empirical data recorded from the cuneate nucleus (CN) and primary somatosensory cortex (S1) of monkeys performing active and passive arm movements. Parts of this figure were created with BioRender.com

Building on this concept, Marin Vargas and Bisi et al. developed a normative framework to uncover the computational principles underlying the proprioceptive pathway. Using a multifaceted strategy, they integrated several techniques: (i) simulating proprioceptive inputs through advanced musculoskeletal modeling, (ii) optimizing neural network models based on hypotheses representing distinct goals of proprioceptive processing, and (iii) predicting neural activity in the CN and S1 of monkeys performing active and passive arm movements (Fig. 1).

Training adequate models of proprioception requires a diverse and extensive repertoire of movements and their corresponding muscle spindle signals, which are difficult to obtain due to the anatomical location of the proprioceptive pathway. To address this challenge, Marin Vargas and Bisi et al. generated a large-scale dataset of synthetic muscle spindle signals using a sophisticated three-dimensional musculoskeletal arm model. This dataset provided the necessary foundation for training thousands of neural networks based on various architectures and learning algorithms, each designed to effectively model time-series data.

Building on this foundational dataset, the authors used these neural networks to explicitly test candidate hypotheses about proprioceptive processing. They evaluated 16 distinct hypotheses that have been proposed over decades of proprioceptive research, covering areas such as kinematic state estimation, action recognition, sensorimotor control, and efficient coding. Each hypothesis was formulated as a computational objective for which a set of neural networks were specifically trained.

To test which task optimization most accurately reflects proprioceptive processing in the brain, the authors evaluated their models by using the learned representations to predict extracellular electrophysiological recordings in CN and S1 of monkeys performing both active and passive small arm movements in a center-out reaching paradigm. Critically, the task-optimized models were able to generalize from simulated to empirical proprioceptive data, validating the effectiveness of this approach. Furthermore, these models outperformed several control models, including classical linear encoding models and data-driven neural networks trained directly on the empirical proprioceptive data, in predicting neural signal dynamics.

This task-driven modeling approach yielded multiple insights into the computational mechanisms underlying proprioception, highlighting several major findings. First, models optimized for kinematic state estimation of limb position and velocity were most effective at predicting neural activity in CN and S1, underscoring the importance of these coding signals in the proprioceptive pathway. Second, a positive correlation was observed between the models’ effectiveness in solving their computational tasks and their predictive accuracy with actual neural data, emphasizing the role of model architecture and task optimization in developing brain-like representations. Third, task-optimized models significantly outperformed randomly initialized untrained models during active movements, but not during passive movements, suggesting potential top-down modulation of CN and S1 during voluntary movements. Fourth, despite their hierarchical anatomical organization, both CN and S1 were best explained by deep layers of the models. Together, these findings illustrate that kinematic state estimation is a fundamental computational goal of the proprioceptive pathway and reveals critical factors in how proprioceptive signals are processed.

Despite the computational tour-de-force, several questions remain open: (i) While task-optimized models were more effective at explaining neural activity in CN and S1 during active compared to passive movements, it is still unclear how models could adequately represent passive movements. (ii) The experiments limited the monkeys’ workspace to small movements, raising questions about whether the findings generalize to larger, more complex workspaces. (iii) Proprioceptive signals typically co-occur with other sensory signals, such as visual and tactile information. Modeling the integration of these diverse inputs remains an unsolved challenge.

The final question might be the most speculative: Where can all this take us? The normative framework introduced by Marin Vargas and Bisi et al. has the potential to be extended to the integration of multiple sensory domains, such as proprioception, vision, and touch, enhancing our understanding of comprehensive sensory processing. Additionally, the study holds the promise of potential disruptive advancements in the field of neuroprosthetics. Despite significant progress in controlling robotic arms, until very recently, these movements lacked a crucial feature: sensory feedback. While direct stimulation now allows for the recreation of touch,^5^ effectively simulating the corresponding proprioceptive sensation remains a challenge. The work of Marin Vargas and Bisi et al. represents an important step toward that goal.
